# Low prevalence of hemoperitoneum recurrence in dogs following splenectomy for benign splenic tumors

**DOI:** 10.3389/fvets.2025.1636456

**Published:** 2025-08-25

**Authors:** Brooke T. Fourthman, Samuel D. Stewart, Heather Wilson-Robles, Chand Khanna, Jacob Cawley

**Affiliations:** ^1^Las Vegas Veterinary Specialty Center, Las Vegas, NV, United States; ^2^Ethos Discovery, Sorrento Valley, CA, United States

**Keywords:** hemoperitoneum, recurrence, splenectomy, benign, dog

## Abstract

**Introduction:**

The purpose of this study is to describe the outcomes and prevalence of hemoperitoneum recurrence in dogs presumptively cured following splenectomy for spontaneously ruptured benign splenic lesions.

**Methods:**

A retrospective analysis of a cohort of 83 client-owned dogs with spontaneous hemoperitoneum due to a histologically benign, bleeding splenic lesion was performed. Medical records of dogs with ruptured benign splenic tumors presenting with hemoperitoneum were reviewed, in addition to owner follow-up, to determine if subsequent hemoperitoneum events occurred. Data were analyzed using statistical software(GraphPad Prism 10.1.2).

**Results:**

A total of 59 patients (71%) were alive at the end of the follow-up period (median follow-up duration of 375 days; range: 128–1,062),with no new concerns related to previous splenectomy or hemoperitoneum. Of the 59 dogs, 6 died and 18 dogs (28.9%) were euthanized during the follow up period. Recurrent hemoperitoneum was identified in three dogs at 40, 68,and 385 days postoperatively, associated with a new liver lesion, an abdominal lesion of unclear origin, or hepatic nodules as the reason for the rebleeding events. Additional sectioning of the initial lesions was not performed.

**Discussion:**

Second hemoperitoneum events occurred, but were uncommon, accounting for 3.6%of cases in this study. Reasons for recurrent spontaneous hemoperitoneum may include the development of a new lesion, the presence of a secondary non-splenic lesion that was unidentified during preoperative staging or abdominal exploratory, or histopathologic misdiagnosis of the original lesion.

## Introduction

Canine spontaneous hemoperitoneum (SH), or the accumulation of blood within the peritoneal cavity, is a common reason for canine emergency presentations (1). The spleen is the most common source of hemorrhage in dogs, regardless of breed, accounting for 43–90% of SH cases (2, 3). Benign or malignant lesions can contribute to splenic hemorrhage. Hemangiosarcoma (HSA), an aggressive cancer of vascular origin, poses a high risk for recurrent SH post-splenectomy (2, 4–6). Retrospective studies may overestimate the percentage of malignant splenic lesions identified in cases of SH. In contrast, prospective studies have found a higher percentage of benign splenic lesions, even in older large-breed dogs, ranging from 35.7 to 47.8% (5, 7).

Benign splenic lesions have previously been reported to account for 35.7–39% of canine splenic SH cases and include splenic hematoma, nodular hyperplasia, splenic infarct, extramedullary hematopoiesis, myelolipoma, hemangioma, red pulp congestion, splenic abscess, and fibrosis (4, 5, 7). Historically, patients undergoing splenectomy for benign ruptured splenic lesions had a good long-term prognosis, free from progressive disease or recurrent SH (4). A recent study evaluating premature death in patients who had benign splenic pathologies identified hemorrhagic pericardial or peritoneal effusion in 66.7% of the nine patients (8). In this study, patients experiencing adverse outcomes had a median survival time of 49 days despite benign histopathology (8).

Millar et al. reported recurrent SH in 5.6% of their total study population and recurrent bleeding (either SH or pericardial effusion) in 11.1% of their population following splenectomy for benign ruptured splenic masses (8). All of the dogs who exhibited recurrent bleeding in Millar’s study were originally diagnosed with one of the following: splenic hematoma, splenic nodular hyperplasia, or splenic hemangioma. Of the three dogs that presented with pericardial effusion, one was diagnosed with a mass at the right atrioventricular groove. Necropsy was performed in one dog that presented with SH, and hemangiosarcoma was diagnosed at that time. The remaining three dogs in this study, who had a benign lesion with premature death, had disseminated intravascular coagulopathy, sudden collapse, and multiorgan dysfunction syndrome as their cause of death. Phipps et al. identified that thrombocytosis and abnormal thromboelastography parameters are common following splenectomy for both malignant and benign lesions, suggesting that postoperative hypercoagulability risk may increase the risk of complications (9). The purpose of this study is to better define second bleeding events in a prospectively accrued cohort of dogs with benign etiologies. We hypothesize that second hemoperitoneum events in patients undergoing splenectomy for benign pathology are uncommon, occurring in less than 5% of cases.

## Materials and methods

Canine patients enrolled in the prospective nationwide clinical trial (Ethos Precision Medicine Umbrella Study for Hemangiosarcoma [Ethos-PUSH]) were evaluated for inclusion in this study. Ethos-PUSH cases were enrolled in 25 participating hospitals nationwide and underwent routine splenectomy for SH secondary to a ruptured splenic tumor. Dogs were required to have no evidence of metastasis on thoracic radiographs prior to surgery, which were reviewed by the admitting clinician. The medical records of dogs enrolled in Ethos-PUSH from October 2020 to July 2023 were reviewed. Dogs that underwent splenectomy and were subsequently confirmed to have a histologically benign splenic lesion were eligible to be included in the current retrospective study. Dogs with malignant pathology or inconclusive histopathology reports were excluded. All splenic lesions were evaluated by a boarded pathologist at a regional commercial laboratory.

Referring veterinarians of the included dogs were contacted to obtain up-to-date medical records. The collected data included current clinical status, date of last follow-up, date of death, and cause of death or reason for euthanasia, if applicable. Pet owners were contacted via phone if recent follow-up data were not available through the referring veterinarian (i.e., >6 months since last follow-up). Pet owners were surveyed for newly identified problems, suspected cause of death or reason for euthanasia if applicable, and knowledge of any clinical signs or recent diagnostics consistent with hemoperitoneum. Dogs of pet owners who did not respond or were euthanized for unknown reasons were excluded.

Data were analyzed using statistical software (GraphPad Prism 10.1.2). Normality for each continuous variable was assessed using the Shapiro–Wilk tests. None of the continuous variables were normally distributed; therefore, descriptive statistics are reported using medians and ranges.

## Results

Of 248 total cases enrolled in Ethos-PUSH at the time of analysis, 94 met the inclusion criteria for this study. Seven patients were lost to follow-up, and four patients were euthanized for unknown reasons and were therefore excluded from evaluation, leaving 83 patient records for review (Table 1). A total of four dogs (4.8%) diagnosed with a benign etiology associated with splenic SH had evidence of recurrent bleeding post-splenectomy, including three with hemoperitoneum and one with hemothorax.

**Table 1:** Number of cases included and excluded.

Cases	Number of dogs
Dogs with benign histopathologies	94
Dogs lost to follow-up	7
Euthanized for unknown reasons	4
Total dogs included	83

The most common breeds presented were mixed breeds (*n* = 14) and Labrador Retrievers (*n* = 11) (Table 2). The median age at the time of SH presentation was 10 years (range: 5–15), with the median weight being 27.5 kg (range 7.1–77). The most common histopathologic diagnosis was nodular hyperplasia in 78.3% (*n* = 65) of patients, followed by myelolipoma in 8.4% (*n* = 7) and hematoma in 4.8% of patients (*n* = 4). There was one patient with each of the following diagnoses: benign nodular lymphoid hyperplasia, extramedullary hematopoiesis, hemangioma, hematopoietic hyperplastic nodule, nodular extramedullary hematopoiesis, red pulp congestion, and splenic abscess.

**Table 2:** Sex and breed demographics of the included dogs.

Sex	Number of dogs
Male Castrated	45
Female Spayed	35
Male Intact	3

Breeds	Number of Dogs
Mixed Breed	14
Labrador Retriever	11
French Bulldog	5
English Bulldog	5
Golden Retriever	4
Pitbull	4
German Shepherd Dog	4
Boxer	4
Dachshund	3
Siberian Husky	2
Pembroke Welsh Corgi	2
American Staffordshire	2
Goldendoodle	2
Plott Hound	2
Labradoodle	1
Australian Cattle Dog	1
Yorkshire Terrier	1
Bull Terrier	1
Bernese Mountain Dog	1
Swiss Mountain Dog	1
Doberman	1
Australian Shepherd	1
Chesapeake Bay Retriever	1
Boerboel	1
West Highland Terrier	1
Shiba Inu	1
Beagle	1
Weimaraner	1
Cockapoo	1
English Mastiff	1
Gordon Setter	1
Wheaten Terrier	1
Shih Tzu	1

Recurrent hemoperitoneum was identified in three dogs (3.6% of the total population) at 40, 68, and 385 days postoperatively, with either a liver lesion, cranial abdominal lesion, or multiple hepatic nodules noted as the likely etiology for the hemorrhage. The dog with hemothorax died 4 days postoperatively. Necropsy was not performed on any of the cases of recurrent bleeding, and nodular hyperplasia was the histopathologic diagnosis in all these cases. The cause of hemothorax was not determined, as it was identified following cardiac arrest and cardiopulmonary resuscitation (CPR). Fifty-nine patients (71%) were alive at the end of the follow-up period and presumed to be disease-free with a median follow-up time of 375 days (range: 128–1,062). None of these dogs were reported to have any new concerns related to their previous splenectomy and initial SH event. Six dogs (6.9%) died without euthanasia, with a median of 4 days (range: 2–204 days) following splenectomy for nodular hyperplasia (*n* = 5) and myelolipoma (*n* = 1). Necropsy was not performed in any of these dogs. Four of these dogs were suspected by the attending clinician to have died secondary to a thromboembolic event or fatal arrhythmia, with hemorrhage ruled out on point-of-care ultrasound. Three of these dogs died acutely at home within 5 days of discharge, and one dog died acutely 2 days postoperatively while hospitalized with an acute onset of respiratory distress. The dog that was arrested in the hospital following respiratory distress had an unremarkable recheck of the thoracic radiographs shortly prior to arrest. This dog also exhibited ventricular tachycardia during hospitalization but was well controlled on mexiletine at the time of arrest. The three dogs who died acutely at home were all receiving antiarrhythmics (either sotalol or mexiletine) for ventricular premature complexes that were well controlled prior to discharge. One dog died of upper airway obstruction secondary to a cervical lesion 204 days postoperatively. More than 50% of the population diagnosed with benign pathology as the cause of their splenic rupture were alive at the end of the follow-up period; therefore, the median survival time for all dogs post-splenectomy for spontaneous hemoperitoneum was not reached.

Eighteen (21.7%) dogs were euthanized at a median duration of 328 days (range: 67–844 days) following surgery. The most common reason for euthanasia was the diagnosis or suspicion of a new neoplastic condition (*n* = 9, 50%). These included: cutaneous lymphoma, advanced-stage histiocytic sarcoma, renal lymphoma, T-cell lymphoma, a right atrial lesion, an abdominal lesion of unknown origin, a thoracic lesion, a nasal lesion, and an anal gland tumor. The right atrial lesion was diagnosed 273 days after splenectomy. The second most common reason for euthanasia was mobility-related difficulties, including osteoarthritis, tetraparesis, and pelvic limb ataxia (*n* = 5, 27.8%). One patient was humanely euthanized following a transfusion-related reaction during the treatment of Babesia.

Four dogs were presented following discharge with recurrent thoracic (*n* = 1) or abdominal (*n* = 3) bleeding, each of whom had preoperative thoracic radiographs previously performed. The dog with hemothorax was an 11-year-old female spayed Plott Hound who presented 3 days postoperatively with pale gums and panting. During initial stabilization, the dog went into cardiac arrest, and CPR was initiated. During resuscitative efforts, thoracic ultrasound identified pleural effusion, which was sampled and consistent with non-clotting hemorrhagic effusion. The return of spontaneous circulation was not achieved, and the owners elected to discontinue CPR.

Three additional dogs presented with recurrent hemoperitoneum. A 9-year-old castrated male German Shepherd Dog was presented 40 days post-splenectomy for a rehabilitation appointment at which time pale gums were noted. A point-of-care abdominal ultrasound identified abdominal effusion with a packed cell volume of 39%. The peripheral packed cell volume in circulation at that time was 31%. Thoracic radiographs did not identify any new abnormalities. An abdominal ultrasound identified a smooth, homogenous lesion in the mesentery with small, smooth hepatic nodules in addition to abdominal effusion. A cytology of the mesentery lesion identified a few normal hepatocytes. The dog was started on Yunnan Baiyao, and whole body computed tomography was scheduled for 5 days later, which identified new liver nodules, the largest measuring 2.3 × 2.7 cm. Continued monitoring and medical management were performed. The dog was presented 67 days post-splenectomy for a 48-h history of lethargy and decreased appetite. A recheck abdominal ultrasound identified progressive abdominal effusion and liver nodules. The owner elected to proceed with humane euthanasia. A necropsy was not performed.

A second dog with a secondary SH event was a 10-year-old male castrated Goldendoodle that presented 68 days postoperatively for lethargy and weakness. Point-of-care abdominal ultrasound identified abdominal effusion, which was confirmed to be hemorrhagic, as well as a suspected liver lesion. Based on these findings, the owner elected euthanasia. A necropsy was not performed.

The remaining dog with a secondary SH event was a 12-year-old female spayed Labrador Retriever. She presented 385 days postoperatively with weakness and inability to walk. A point-of-care abdominal ultrasound identified abdominal effusion, confirmed to be hemorrhagic, as well as a large cranial abdominal lesion. Advanced imaging and surgical consultation were discussed with the owners, but euthanasia was ultimately elected. A necropsy was also not performed in this case.

## Discussion

Secondary SH events are uncommon following splenectomy for benign etiologies, occurring in only 3.6% of the 83 dogs in this cohort. Two of the three dogs that exhibited a secondary SH event displayed clinical signs and were euthanized within 68 days of surgery. The short postoperative period prior to SH in these two cases suggests either the presence of an additional neoplastic lesion that was not identified during staging or abdominal exploratory or failure to identify a malignant neoplasm on histopathology. SH was identified in the remaining dog 385 days postoperatively. The prolonged period between SH events in this dog could be suggestive of different etiologies. One additional dog had a secondary bleeding event outside of the abdomen 4 days post-splenectomy despite unremarkable preoperative thoracic radiographs.

In this study, the most commonly affected breeds included mixed breeds and Labrador Retrievers, with a median age of 10 years. These findings are consistent with previous studies evaluating SH, which identified mixed breeds, Golden Retrievers, Labrador Retrievers, and German Shepherd Dogs as the most commonly affected (2, 3, 7). SH secondary to a splenic lesion is more commonly identified in large-breed dogs (2). While large-breed dogs are more likely to be diagnosed with hemangiosarcoma than small-breed dogs (2), this study demonstrated that they are also diagnosed with benign etiologies in approximately 40% of cases. The most common histopathologic diagnoses in this study were nodular hyperplasia (78.3%) and myelolipoma (8.4%).

Over 70% of the study population was alive at the time of follow-up. None of these cases reported any new concerns related to their previous SH event and were presumed to be cured. In this study, the median follow-up time was 375 days postoperatively. A small percentage of dogs (6.9%) in this study died within a median duration of 4 days postoperatively, with a suspected thromboembolic event or fatal arrhythmia being the most common cause of death. Recurrent hemorrhage was ruled out on point-of-care ultrasound in these cases. In three of these four cases, the dog was discharged on an antiarrhythmic medication (either sotalol or mexiletine) for ventricular arrhythmias that were well controlled prior to discharge. None of these patients had thromboelastography performed or received anticoagulant medications postoperatively. Phipps et al. determined that, following splenectomy, dogs may be hypercoagulable and at greater risk for thrombotic conditions (9). This study did not report any clinically significant ramifications associated with the identified thrombocytosis or abnormal thromboelastography values, but anticoagulants may be recommended in the future in patients at risk for hypercoagulopathies.

A little over 20% of the study population was euthanized within a median of 328 days postoperatively, most commonly due to a new neoplastic condition (50%). This included one dog with a right atrial hemangiosarcoma identified at necropsy 273 days following splenectomy. The second most common reason for euthanasia was mobility-related problems (27.8%), both orthopedic and neurologic in origin.

Three dogs experienced secondary SH events resulting in euthanasia in each case. SH occurred 40, 68, and 385 days postoperatively, and in each case, hemorrhagic effusion was identified through point-of-care ultrasound. A new abdominal or liver lesion was identified either on point-of-care ultrasound or additional imaging. The diagnosis of secondary SH with new abdominal or liver lesions suggests that either a malignant lesion was missed during histopathologic evaluation or a secondary lesion was not identified during preoperative staging or abdominal exploration. Additional sectioning of the initial lesions was not performed and may be considered in the future to improve confidence in a benign histopathologic diagnosis. The initial screening and diagnostic criteria were standardized in the initial prospective clinical trial and included preoperative thoracic radiographs. Suspicion for an unidentified primary HSA lesion may justify advanced imaging such as computed tomography (CT). CT was not required in patients for inclusion in the prospective clinical trial that was used for this retrospective study. CT has previously been investigated for its diagnostic ability to distinguish between malignant and benign pathology in non-ruptured splenic masses and to identify evidence of metastasis; in this study, there was a significant difference in attenuation between malignant and benign lesions, making it a useful preoperative imaging modality for non-ruptured splenic masses (10). Preoperative use of CT was evaluated in a retrospective case series for non-traumatic hemoperitoneum and indicated that it was not a reliable indicator of malignancy in these cases (11, 12). However, to the authors’ knowledge, this has not yet been investigated prospectively.

Limitations of this study include the retrospective nature, resulting in variability of medical record data and diagnostics performed, both from tertiary referral hospitals and primary care veterinarians. Of the four patients who died in the short-term postoperative period, three died at home, preventing the identification of a definitive cause of death, but fatal arrhythmia or thromboembolic events were suspected in these cases. Dogs undergoing splenectomy for SH are at risk for thromboembolic events postoperatively, accounting for over 30% of deaths in dogs that did not survive discharge in the study by Wendelburg et al. (13). None of the patients surviving to the end of the follow-up period reported any concerns related to their initial SH event. However, due to the previous benign diagnosis, most patients did not have follow-up imaging to definitively rule out a new lesion or effusion. For those cases with secondary SH events, histopathologic evaluation of the new abdominal lesions was not available. Lack of necropsy in cases of recurrent SH is an additional limitation of this study.

Splenectomy for SH secondary to benign etiologies has a good prognosis, and second SH events are a complication with low prevalence; 3.6% in this study. The majority of patients who died during the follow-up period died or were euthanized due to unrelated causes. Veterinarians can be confident that the pathology of the spleen will correctly identify benign lesions in the vast majority of cases, allowing for better prognostication in dogs with benign lesions. Because histopathologic evaluation of the new lesions identified in the three cases of recurrent SH in this study was not performed, the etiology of these second SH events is unknown. Possible causes of second SH in these cases may include hemorrhage from new lesions, hemorrhage from unidentified pre-existing lesions, or hemorrhage from histopathologic misdiagnosis at the time of surgery. Further evaluation of the underlying etiology of second SH events following splenectomy for benign histopathologies is needed.

## Data Availability

The raw data supporting the conclusions of this article will be made available by the authors, without undue reservation.
